# Potential of Terpenoids and Flavonoids from Asteraceae as Anti-Inflammatory, Antitumor, and Antiparasitic Agents

**DOI:** 10.1155/2017/6196198

**Published:** 2017-07-12

**Authors:** Valeria P. Sülsen, Emilio Lizarraga, Nilufar Z. Mamadalieva, João Henrique G. Lago

**Affiliations:** ^1^Departamento de Farmacología, Cátedra de Farmacognosia, Facultad de Farmacia y Bioquímica, Universidad de Buenos Aires, Buenos Aires, Argentina; ^2^Universidad de Buenos Aires, CONICET, Instituto de Química y Metabolismo del Fármaco (IQUIMEFA), Buenos Aires, Argentina; ^3^Instituto de Fisiología Animal, Fundación Miguel Lillo y Facultad de Ciencias Naturales e Instituto Miguel Lillo, Universidad Nacional de Tucumán, Tucumán, Argentina; ^4^Institute of the Chemistry of Plant Substances, Academy of Sciences of Uzbekistan, 100170 Tashkent, Uzbekistan; ^5^Federal University of ABC, Center of Natural Sciences and Humanities (CCNH-UFABC), Santo André, SP, Brazil

Asteraceae (formerly known as Compositae) is one of the largest families of higher plants, with more than 1700 genera and approximately 24000 species, which grow in varied environments [1]. The economic importance of the Asteraceae family has been described and, for centuries, several species of this family have been used for medicinal and food purposes [2].

Over the last decades, different species from this family have been studied due to the great variety and amount of bioactive compounds they synthesize. Among them, terpenoids and flavonoids stand out because of their biological activities and potential health benefits.

Terpenoids constitute the largest class of natural products derived from isoprene (C5) units joined head-to-tail or tail-to-head, among other possibilities. They are classified as hemiterpenes (C5), monoterpenes (C10), sesquiterpenes (C15), diterpenes (C20), sesterpenes (C25), triterpenes (C30), tetraterpenes (C40), and polyterpenes (>C40). They can be found in numerous living organisms, especially plants, fungi, and marine animals. Terpenoids are of great interest due to the broad range of biological activities reported such as cancer preventive effects and analgesic, anti-inflammatory, antimicrobial, antifungal, antiviral, and antiparasitic activities [3].

Flavonoids are hydroxylated phenolic compounds that are present in plants and occupy a special place among secondary metabolites. They are classified into different classes, with flavones, flavonols, flavanones, catechins, isoflavones, and anthocyanidins being the most common. Similar to terpenoids, they also present a wide range of biological activities. These compounds have been demonstrated to have protective effects against many infectious and degenerative diseases such as cancer, among other important pharmacological activities such as antioxidant and anti-inflammatory activities [4, 5].

Many of these bioactive compounds and their derivatives either are already being used to treat diseases or are under study in preclinical and clinical trials. The sesquiterpene lactones artemisinin and arglabin, isolated both from* Artemisia* species, are approved drugs for the treatment of human malaria and cancer, respectively. The anticancer drug paclitaxel is also a terpenoid compound used nowadays against several types of cancer [6]. Among flavonoids, the flavone quercetin is currently being assessed in clinical trials on prostate cancer and its primary prevention [7].

This special issue offers original research contributions related to the evaluation of antiparasitic and cytotoxic extracts by means of screening processes, the evaluation of the effect of extracts on inflammation and the detection of their bioactive compounds, and the assessment of the anti-inflammatory and cytotoxic activities of terpenoids and flavonoids as well as those formulae containing such compounds, together with attempts to gain an insight into the possible mechanism of action of these groups of substances.

Considering the biological and pharmacological activities of terpenoids and flavonoids and the importance of these metabolites as potential lead compounds, we decided to include in this special issue some research articles describing the activity of these groups of compounds isolated from species belonging to families other than Asteraceae.
